# Application of microalgae as natural colorant for pastry and confectionary products

**DOI:** 10.1002/fsn3.4394

**Published:** 2024-10-17

**Authors:** Tatiana Pereira, Sónia Barroso, Filipa R. Pinto, Frederica Silva, Paula Teixeira, Susana Mendes, Maria M. Gil

**Affiliations:** ^1^ MARE – Marine and Environmental Sciences Centre/ARNET – Aquatic Research Network ESTM, Polytechnic of Leiria, Cetemares Peniche Portugal; ^2^ MARE – Marine and Environmental Sciences Centre/ARNET – Aquatic Research Network, Faculdade de Ciências Universidade de Lisboa Lisbon Portugal; ^3^ CBQF – Centro de Biotecnologia e Química Fina – Laboratório Associado, Escola Superior de Biotecnologia Universidade Católica Portuguesa Porto Portugal

**Keywords:** bioactivity, clean label, coloring, egg substitute, microalgae, natural

## Abstract

Modern consumers demand the replacement of synthetic colorants with natural alternatives. Microalgae can serve as an alternative source for these colorants since they hold significant amounts of pigments. This study aimed to evaluate the potential of using microalgae biomass and extracts as natural colorants for pastry and confectionary products. The application of different biomass and levels of *Chlorella vulgaris* (White, Honey, and a mixture of both) was evaluated in brioche‐type breads as egg substitute to confer the typical yellow coloration to the product. A mixture of 1% *Chlorella vulgaris* (White: Honey [1:1]) showed potential as egg substitute, having minimal impact on the physical–chemical, microbiological, nutritional, and sensory characteristics of the products. Hydroalcoholic *Tetraselmis chuii* extracts were applied in fondant at a concentration of 0.05%, providing a green coloration with minimal impact on the quality of the product. This study supported the potential of using microalgae, biomass, and extracts, as alternative natural colorants in pastry and confectionary products.

## Graphical abstract

This study evaluated the application of microalgae biomass and extracts as natural colorants for pastry and confectionary products. Chlorella vulgaris biomass was used in brioche‐type breads as egg substitute, to confer the typical yellow coloration to the product. *Tetraselmis chuii* extracts were applied in fondant providing a green coloration with minimal impact on the quality of the product. This study supported the potential of using microalgae, both biomass and extracts, as alternative natural colorants in pastry and confectionary products.

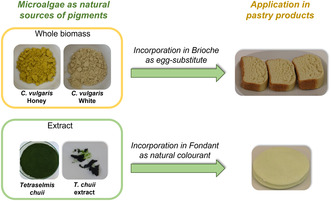

## 1 INTRODUCTION

The modern consumer has been pushing for foods with healthier and cleaner labels, driving the search for novel natural sources of ingredients/compounds able to compete with the synthetic food additives.

Color is one of the most impactful characteristics of foods and can play the deciding card on the moment of the choice to purchase and eat, because color sends important cues about the expected flavor/taste and safety of the products (Spence, 2015). Colorants are used to give, enhance, intensify, and standardize the coloring of foods (Amchova et al., 2015; Ngamwonglumlert et al., 2017). Nevertheless, the possible association of health hazards with the use of synthetic dyes has tarnished their reputation and led to the need to find natural alternatives (Dey & Nagababu, 2022; Ngamwonglumlert et al., 2017). However, natural colorants are still not able to match the low cost, availability, and stability of the existing synthetic colorants (Dey & Nagababu, 2022; Sigurdson et al., 2017). These natural alternatives are known to be more susceptible than their synthetic counterparts to degradation through various natural conditions such as light, temperature, oxygen, and pH (Ngamwonglumlert et al., 2017; Sigurdson et al., 2017). The market value of food colorants was USD 3.13 billion in 2023 and from those 43% was revenue from natural food colorants (Grand View Research, 2024).

On the other hand, current diet trends and allergenicity issues are calling for egg‐reduced or egg‐free alternatives (Yazici & Ozer, 2021). The “egg replacers” market has been valued by Market Data Forecast (2024) at around USD 1.57 billion being estimated to increase 6% annually reaching USD 210 billion in 2029. Nevertheless, replacing eggs in bakery products presents some challenges since they play important roles in the cake dough. Hedayati et al. (2022) reviewed the role of eggs in cakes as well as the effects of using alternative emulsifiers, hydrocolloids, proteins, legumes, seeds, and fruit pulp and pomace on the batter and cake quality. Eggs, yolk and white, contribute to the formation and stabilization of foam, act as emulsifiers, and contribute to the breakdown of fat particles and to the tension at the oil–water interface (Hedayati et al., 2022; Wilderjans et al., 2013). Eggs also play a role in providing color, flavor, in leavening, volume, texture, and nutritional value, among others (Hedayati et al., 2022; Peris et al., 2019).

Microalgae have been used as natural supplements and food colorants (Schüler et al., 2020). Their rich nutritional composition is the main reason for the widespread commercialization of microalgae, such as *Chlorella vulgaris* and *Arthrospira platensis* (Spirulina), as dietary supplements. More recently, *Tetraselmis chuii* has been added to the restricted group of approved microalgae to be used in food products (Regulation (EU) 2017/2470, 2017). In terms of color, microalgae present a great deal of pigments that can serve as natural colorants such as chlorophyll, carotenoids (β‐carotene and astaxanthin), and phycobiliproteins (phycoerythrin and phycocyanin) (Luzardo‐Ocampo et al., 2021).

The use of the whole biomass presents the advantage of not only providing coloration but also enriching the food with the nutrients available in the microalgae. However, the characteristic algae taste provided by these ingredients can be a major drawback in the commercialization of such products. The distinct flavor of microalgae has been previously associated with not only their chlorophyll content but also the presence of some volatile compounds such as linear aldehydes, terpenes/isoprenoids, and sulfuric compounds (Schüler et al., 2020; van Durme et al., 2013). Thus, major efforts have been made to reduce the taste of microalgae with the development of microalgae with reduced chlorophyll content such as *Chlorella vulgaris* Smooth, White, and Honey (Allma, 2021; Pereira et al., 2024; Schüler et al., 2020). A consumer study on microalgae‐based bread and beer has shown that environmentally conscious and innovative consumers are more willing to pay for these products (Maehle & Skjeret, 2022).

Considering this, the main objective of this study was to evaluate the application of *Chlorella vulgaris* (White and Honey) biomass and *Tetraselmis chuii* extracts as natural colorants in representative confectionary products, namely Brioche and Fondant. In brioche, it is intended to replace the eggs and provide the color with the addition of microalgae biomass while in fondant, the color is to be provided by microalgae extracts as replacement of synthetic colorants. Moreover, the effect of adding microalgae on physical–chemical properties, nutritional value, and microbial stability was also evaluated.

## 2 MATERIALS AND METHODS

### 2.1 Microalgae

*Chlorella vulgaris* biomass in the colors yellow (*C. vulgaris* Honey) and white (*C. vulgaris* White) (Allma, 2021) and *T. chuii* were kindly provided by Allma from Allmicroalgae (Lisbon, Portugal).

### 2.2 Materials

The bakery ingredients were purchased from a local market.

The following reagents were used in this study: Chloroform (99.2%, VWR, Fontenay sous Bois, France), methanol (99.9%, Carlo Erba, Val‐de‐Reuil, France), n‐hexane (95.0%, Carlo Erba), sodium sulfate anhydrous (≥ 99.0%, Honeywell, Seelze, Germany), sulfuric acid (95.0–97.0%, Honeywell), sodium chloride (99.9%, Biochem, Cosne‐Cours‐sur‐Loire, France), and ethanol (99.5%, Aga, Prior Velho, Lisbon, Portugal).

### 2.3 Preparation of the extracts

*Tetraselmis chuii* extracts were obtained using the procedure described by Kulkarni and Nikolov (2018) for the extraction of chlorophyll from *C. vulgaris*, with some modifications. Dried *T. chuii* biomass (1 g) was stirred with 20 mL of ethanol 80% v/v at room temperature for 30 min. Then, the solution was centrifuged (Eppendorf Centrifuge 5810R, Enfield, CT, USA) at 7500*g* (10 min, 4°C), and the supernatant was separated and stored. The pellet was subjected to a second extraction, following the same extraction procedure. After the second centrifugation, the resulting supernatants were pooled and filtered through a Whatman filter (0.45 μm) to ensure the removal of any residual biomass particles. Supernatants were subjected to spectrophotometric readings at 470, 649, and 664 nm before solvent removal through rotary evaporation. To ensure complete solvent removal, the extracts were frozen at −80°C and subsequently dried and in a CoolSafe 55–4 (Labogene, Allerød, Denmark). The dried extracts were stored at −20°C in sealed falcon tubes covered with parafilm until further antioxidant analysis and application.

Equations ((1), (2), (3)) were used to calculate the concentration of chlorophyll and carotenoids present in the supernatants.

(1) $${\text{Chlorophyll}}_{a}\;\left(\mathrm{\mu g}/\text{mL}\right)=\left(13.36\times A_{664}\right)-\left(5.19\times A_{649}\right)$$

(2) $${\text{Chlorophyll}}_{b}\;\left(\mathrm{\mu g}/\text{mL}\right)=\left(27.43\times A_{649}\right)-\left(8.12\times A_{664}\right)$$

(3) $$\begin{array}{c} \text{Total carotenoids}\;\left(\mathrm{\mu g}/\text{mL}\right)\\ =\frac{\left(1000\times A_{470}-2.13\times \text{Chlorophyll}\;a-97.64\times \text{Chlorophyll}\;b\right)}{209} \end{array}$$

The pigment results are presented as mg/g. The extraction yield was also calculated (Equation 4) and expressed as %.

(4) $$\text{Extraction yield}\;\left(\%\right)=\frac{\text{Dried extract}}{\text{Initial sample}}\times 100$$

### 2.4 Preparation of the pastry products

#### 2.4.1 Brioche‐type bread

The brioche‐type breads, control and eggless brioche, were produced with the ingredients described in Table 1. Three different levels of microalgae incorporation were tested in brioche at a concentration of 1% of weight of microalgae/weight of brioche (1% of *C. vulgaris* White, 1% of *C. vulgaris* Honey, and 1% of a mixture of *C. vulgaris* Honey:*C. vulgaris* White [1:1]). This percentage was defined in preliminary studies that evaluated the amount of algae that could be added without affecting the product's characteristics (data not shown).

**TABLE 1:** Representation of the ingredients used in the production of the brioches with (eggless brioche) and without egg substitution (Control).

Ingredients	Control (g)	Eggless brioche (g)
Wheat flour	180.00	180.00
Baker's yeast	3.00	3.00
Salt	0.25	0.25
Sugar	20.00	20.00
Half‐skimmed Milk	30.00	30.00
Margarine	50.00	50.00
Whole egg	58.00	–
*C. vulgaris* White^a^	–	1.60
*C. vulgaris* Honey^a^	–	1.60
Water^b^	–	35.00
Total	341.3	321.5

^a^ In the eggless brioche with 1% *C. vulgaris* White or 1% *C. vulgaris* Honey, 3.2 g of the algae was added.

^b^ The amount of water used in the eggless brioche was the equivalent to the contribution of the egg whites (~ 60%; Hedayati et al., 2022; Wilderjans et al., 2013).

The ingredients (yeast, flour, sugar, salt, and eggs) were mixed in a food processor (Bimby, Vorwerk, Carnaxide, Portugal) at room temperature using a low speed (1) for 5 min, followed by 10 min at 2.5 speed. In the eggless brioche, the eggs were replaced by water and microalgae. Afterward, melted butter and milk were added, and the dough kneaded for an additional 10 min, until the dough came away from the sides of the bowl. The dough was transferred to a slightly greased and lightly floured tray and left to leaven for 1.5 h covered with a cloth. The brioche was baked at 180°C (controlled with an oven thermometer) for 19 min turning the overhead heat in the last 3 min until the surface acquired a golden‐brown coloration. The baked brioche was left to cool on a wire rack for 90 min.

After cooling, the samples were weighed, measured, and sliced for analysis of pH, *a*_w_, color, moisture, and texture. A portion of the samples was freeze‐dried and stored in closed plastic bags at −20°C until subsequent nutritional analysis.

Three independent batches were produced on the same day.

#### 2.4.2 Fondant

The ingredients used in the production of the fondant, with and without *T. chuii* extracts, are shown in Table 2. Various percentages of the extracts were tested until reaching a level of extract that provided color with minimum algae flavor in the product (data not shown). The percentage was set at 0.05% (weight of extract/weight of fondant).

**TABLE 2:** Ingredients used in the fondant with and without the *T. chuii* extract.

Ingredients	Control (g)	Fondant with extract (g)
Powdered sugar	78.2	78.2
Corn syrup	7.8	7.8
Gelatine	2.1	2.1
Water	10.4	10.4
Lemon juice	1.5	1.5
*Tetraselmis chuii* extract	–	0.05
Total	100.0	100.0

The fondant was prepared using powdered sugar, unflavored gelatine (Vahiné, Sabadell, Spain), water, and corn syrup (Cem porcento, Samora Correia, Portugal). The extract was first dissolved in ethanol 99.5% v/v (5 mL) and added to a fraction of the powdered sugar. The sugar was incubated overnight in an air oven (Memmert, Schwabach, Germany) at 25°C (50% ventilation) until the ethanol had completely evaporated, and then macerated with a mortar and pestle. Gelatine was dissolved in water and heated at 60°C in a water bath. Once dissolved, the gelatine was mixed with the corn syrup and lemon juice. The colored powdered sugar was placed in a food processor (Bimby) and mixed with the solution at speed 3 until a smooth and malleable structure was achieved (<30 s). Maize starch was used to aid in the molding process.

For color and texture analysis, circles with 6 cm of diameter and 0.5 cm of thickness were prepared. The remaining fondant was stored in sealed bags at 4°C until further analysis.

For each recipe (control fondant and fondant with extract), three independent replicates were prepared on the same day.

### 2.5 Physical–chemical analysis

#### 2.5.1 Color analysis

Color analysis was performed using a Konica Minolta colorimeter (Chroma Meter CR‐400, Japan) using a 2‐degree standard observer and a D65 illuminant. The results were presented as CIELab coordinates, including *L* (lightness, black–white, 0–100), a* (green–red, −60–60), and b* (blue–yellow, −60–60) parameters (Pereira et al., 2024).

For the brioche samples, the color was analyzed in three slices from the middle, and measurements were taken at four different places in the dough, six different places in the baked crust, and four different places in the crumb. All readings were performed five times.

In the cases of fondant samples, the readings were taken at two distinct places within four circles for each formulation (control and fondant with extract). Each reading was repeated at least five times.

The parameter Δ*E* (color differences) was calculated using Equation 5:

(5) $$\Delta E=\sqrt{\Delta L{*}^{2}+\Delta a{*}^{2}+\Delta b{*}^{2}}$$

where ΔL*, Δa*, and Δb* are obtained by subtracting the values of the samples from microalgae to the values of the controls (without microalgae).

The color of the fondants was also analyzed after a storage period of 4 months at 4°C.

#### 2.5.2 Texture analysis

The texture analysis was performed within 24 h after the production of the samples. Texture profile analysis (TPA) was obtained with TA‐XTplus texturometer (Stable MicroSystems, Surrey, UK) equipped with a 5 kg load cell.

For the brioche, the measurements were done on 1.5 cm slices using a 10‐mm‐diameter cylindrical probe that pierced 6 mm into the samples (equivalent to 40% strain) (Correia et al., 2015; Nunes, Graça, et al., 2020). For the fondant, the pieces presented a thickness of 0.5 cm and were analyzed using a 2 mm cylindrical probe that pierced 2 mm into the sample.

All tests were performed at a 1 mm/s test speed with a 5 s waiting time between compressions and using a 30 kg trigger load and a 5 g trigger force. Four readings were performed for each slice of brioche, and five readings were performed for each circle of fondant.

A simplified TPA macro from the software Exponent Connect (Stable Micro Systems, Surrey, UK) was used to extract the values for hardness, resilience, cohesion, and springiness for the brioche, and hardness for the fondant.

#### 2.5.3 pH and *a*_W_

pH was measured using a pH meter (Inolab, WTW, Weilheim, Germany) equipped with a perforation probe, and the *a*_W_ analysis was performed with a hygrometer (HP23‐AW‐A, Rotronic, Bassersdorf, Switzerland) (Khemiri et al., 2020). For the brioche, two samples were crushed to decrease the particle size, and the *a*_w_ was measured in triplicate. For the fondant, three pieces of each formulation were measured.

#### 2.5.4 Proximate composition

Protein was analyzed in an external laboratory using the Dumas method and a nitrogen conversion factor of 6.25 (AOAC 992.23‐1992, 1998; ISO 16634, 2005; Pereira et al., 2024).

Total fat was quantified using the Folch methodology (Folch et al., 1957) with the adaptations described in Pereira et al. (2024). The total fat was extracted from 1 g of sample (dried sample of brioche and fresh sample of fondant) using 0.5 mL of water and 5 mL of chloroform: methanol (2:1), and the mixture was homogenized for 1 min. Then, 5 mL of chloroform: methanol (2:1) was added, and the mixture was homogenized for 5 min. After homogenization (2 min) with 1.2 mL of sodium chloride (0.8%), the solution was centrifuged (6000 rpm, 10 min, 4°C). The lower phase was filtered through a column of hydrophobic cotton and sodium sulfate anhydrous and collected in a pear‐shaped flask. Furthermore, 5 mL of chloroform were added to the supernatant, centrifuged (6000 rpm, 10 min, 4°C), and the lower phase filtered through the column to the pear‐shaped flask. The solvent collected in the flask was evaporated with a rotavapor followed by overnight incubation in an oven. The total fat was calculated through the difference in weight of the pear‐shaped flask.

Moisture was analyzed following the protocol described by Ghendov‐Mosanu et al. (2022). The samples were weighted and incubated overnight in crucibles at 105°C. The moisture was calculated through weight difference.

Ashes were obtained by incubation of the crucibles with the dried samples in a furnace and heating at 535°C for 5 h (NP 2032, 2009). The ashes content was calculated from the differences in weight considering the weight of the initial fresh samples.

Carbohydrates were estimated by subtracting the values of the proteins, fat, moisture, and ashes from 100 g (Khemiri et al., 2020).

#### 2.5.5 Fatty acid profile

The fatty acid (FA) profile was analyzed using the total fat extracted from the brioches dried biomass following the protocol described by Fernández et al. (2015). 10 mg of fat was incubated in a water bath at 80°C for 2 h with 2 mL of methanol with 2% sulfuric acid. 1 mL of milliQ water and 2 mL of n‐hexane were added to the solution, homogenized for 1 min, and centrifuged at 1000 rpm (5 min, 4°C). 1 mL of the upper phase was removed and stored in GC vials until further analysis. The analysis was carried out on a GC‐FID chromatograph (Finnigan trace GC Ultra, Thermo Scientific) equipped with an autosampler (AS 3000, Thermo Electron Corporation) and a TR‐FAME capillary column (Thermo TR‐FAME, 60 m × 0.25 mm ID × 0.25 μm film thickness). The injector (operating in splitless mode) and the detector temperatures were set at 250 and 280°C, respectively. The column temperature was initially set at 100°C for 0.1 min, then raised at 10°C min^−1^ to 150°C and held for 1 min followed by an increase at 5°C min^−1^ to 200°C and maintained for 9 min, and finally raised to 235°C at 2°C min^−1^ and held for 5 min. Helium was used as carrier gas at a flow rate of 1.5 mL min^−1^. Air and hydrogen were supplied to the detector at flow rates of 350 and 35 mL min^−1^, respectively.

The fatty acid profile was determined by comparing the resulting retention times with a 36‐fatty acid standard (Supelco 37 component FAME Mix), and the results were expressed as % of total FA.

### 2.6 Microbiological analysis

For the microbiological analysis, five separate samples of brioches were collected at four different time points during storage at room temperature. The time points included day 1 (24 h after production), day 2, day 3, and day 4.

Similarly, for the monitoring of the fondant, the product was divided into five parts and analyzed every week starting from day 1 (24 h after production) for a total duration of 28 days of storage at room temperature.

In both cases, a control sample without microalgae biomass or extract was used. Each sample (25 g) was homogenized in a stomacher (Interscience, Saint Nom la Brèteche, France) with 225 mL of sterile Ringer's solution (Biokar Diagnostics, Beauvais, France) for 2 min. This was used to prepare appropriate decimal dilutions in Ringer's solution for microbial enumeration along the mentioned time period: Total viable counts at 30°C (ISO 4833‐1, 2013), yeast and molds at 25°C (NP (Norma Portuguesa), 1987), *Bacillus* spp. (Health Protection Agency, 2009), and *B. cereus* (ISO 7932, 2004).

### 2.7 Antioxidant potential

For the antioxidant analyses, ethanolic extracts were produced by adding 1 g of freeze‐dried brioche to 10 mL of ethanol absolute. The mixture was homogenized for 5 min using a vortex and left to rest overnight at 4°C in the dark. The extracts were obtained after 2 cycles of 15 min centrifugation (8000 *g*) at 4°C. For the *T. chuii* extracts, the extraction was performed on 0.34 g of dried extracts with a final concentration of 0.1 g/mL following the methodology described in section 2.7.

The total phenolic compounds were obtained using the Folin–Ciocalteu method, as described by Singleton and Rossi (1965) and Waterhouse (2003) with some modifications. In an Eppendorf, 10 μL of the extract was diluted with 790 μL of distilled water and 50 μL of Folin–Ciocalteu reagent. For the blanks, the Folin–Ciocalteu reagent was replaced by distilled water. The solution was placed in the dark for 2 min (RT). After the addition of 150 μL of 20% sodium carbonate (Na_2_CO_3_), the samples were incubated in the dark at room temperature for 1 h. The samples were transferred to a 96‐well microplate, and the absorbance was measured at 755 nm in a microplate spectrophotometer (Epoch 2, BioTek, United States). Different concentrations of gallic acid (1, 0.3, 0.1, 0.03, and 0.01 mg/mL) were used as reference standards, and the results are presented as mg of gallic acid equivalents/g of sample (mg GAE/ g).

The ability to reduce the DPPH radical was evaluated following the method described by Brand‐Williams et al. (1995) with modifications. 10 μL of the extracts was added to 990 μL of a methanolic solution of DPPH at 0.1 mM and left to react for 30 min in the dark at room temperature. The samples were transferred to a microplate, and the absorbance was measured at 517 nm. Blanks of the samples were prepared using methanol instead of DPPH, and controls were made using ethanol absolute (the solvent of the extracts) in place of the extracts. The ability to reduce DPPH was calculated using Equation (6):

(6) $$\begin{array}{c} \text{Ability to reduce free radicals}\;\left(\%\right)=\\ \;\left(1–\left[A_{\text{sample}}-A_{\text{blank}}\right]\right)/\left(A_{\text{control}}\right)\times 100 \end{array}$$

Ascorbic acid was used as reference standard at 1, 0.75, 0.50, 0.20, and 0.02 mM, and the results are expressed as μmol of ascorbic acid equivalents /g of sample (μmol AAE/ g).

The ferric‐reducing ability of the extracts was analyzed using the methodology described by Benzie and Strain (1996) with modifications. Acetate buffer at 300 mM (pH 3.6) was prepared using sodium acetate (3.1 g/L) and acetic acid (16 mL/L), and the pH was set to 3.6 using NaOH or HCl. The solution of TPTZ was produced by dissolving 10 mM of TPTZ in a solution of 40 mM of HCl. Additionally, the ferric solution (20 mM) was produced by dissolving FeCl_3_.6H25 μL of extract was added to 975 μL of the ferric solution of TPTZ and left in the dark at room temperature for 4 h. The samples were transferred to a microplate, and the absorbance was measured at 593 nm. Blanks of the samples were prepared using water in place of the ferric solution of TPTZ, and different concentrations of ascorbic acid (1, 0.75, 0.50, 0.20, and 0.02 mM) were used as reference standards. The results are presented as μmol AAE/ g.

### 2.8 Sensory analysis

Preliminary consumer acceptance analysis was done using the eggless brioche as a way to evaluate its potential to be commercialized. This analysis was performed by 43 persons between 10 and 65 years, 58% female and 42% male. The analysis was performed in individual sensory booths, evaluating the brioche's color, aroma, taste, texture, and global appreciation using a five‐point hedonic scale (1 – dislike extremely, 2 – dislike, 3 – neither like nor dislike, 4 – like, and 5 – like extremely). The results were composed of the average of each sensory attribute. The participants were also asked to rate their purchase intent.

### 2.9 Statistical analysis

All analyses were done in triplicate and, when applied, the results are expressed as means ± standard deviation. The color differences of the microalgae additions were compared using the Kruskal–Wallis non‐parametric test, followed by the multiple comparison Games–Howell test (Zar, 2010). The comparison of the color differences between the control brioche and the remaining brioches was performed using a Dunnett's test. Differences between the physical–chemical (weight loss, pH, *a*_W_, texture, and color) and nutritional (moisture, ashes, protein, fats, fatty acids, carbohydrates, and energy) results, when comparing the control brioche with the brioche with microalgae and the control fondant with the extract fondant, were assessed using a Student's *t*‐test (Zar, 2010). All assumptions inherent to the performance of the *t*‐test (namely, normality of data and homogeneity of variances) were validated. Whenever the requirements were not met, the Mann–Whitney U test was used. All statistical analyses were performed using IBM SPSS Statistics 28 (Copyright IBM Corp. ©1989–2023, Armonk, NY 10504–1722, USA). All results were considered statistically significant at the 5% significance level (i.e., whenever *p*‐value <.05).

## 3 RESULTS AND DISCUSSION

### 3.1 Brioche‐type bread

**FIGURE 1:**
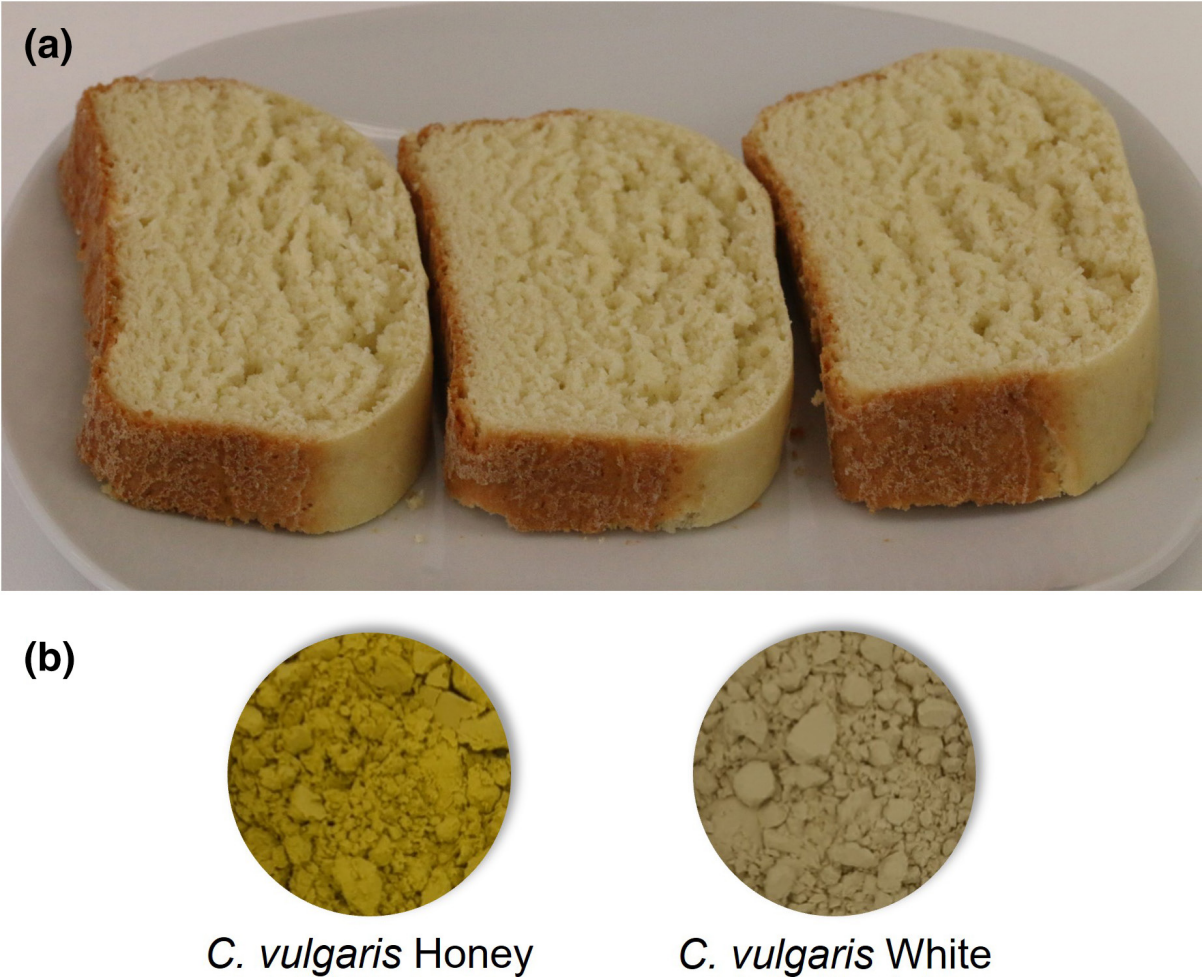
Brioche with 1% *C. vulgaris* (Honey: White) (a) and *C. vulgaris* Honey and White (b).

Brioche‐type bread was chosen as a model for the substitution of egg by *C. vulgaris* (White and Honey) whole biomass. These microalgae were chosen due to their initial coloration that had potential of matching the one provided by the eggs. As mentioned, the objective of this study was to evaluate the potential of using microalgae as a natural color substitute for egg. Considering this, the color parameters L*, a*, and b* were measured to better understand the effect of the different levels of microalgae incorporation when compared with a control‐containing egg (Table 3). Figure 1 shows a representation of the eggless brioche with *C. vulgaris* Honey:White (A), as well as images of the microalgae *C. vulgaris* Honey and White (B).

**TABLE 3:** Results of the color analysis of brioche‐type bread crust and crumb with different microalgae incorporations (1% *C. vulgaris* White [W], 1% *C. vulgaris* Honey [H], and 1% *C. vulgaris* White/*C. vulgaris* Honey [W/H]), and comparison with the crumb of control brioche (C) with egg.

Formulations	Crust			Crumb			Δ*E*	
	L*	a*	b*	L*	a*	b*	Crust	Crumb
C	44.58 ± 8.58^a^	18.54 ± 2.13^a^	30.38 ± 4.82^a^	64.13 ± 2.95^a^	2.17 ± 0.39^a^	22.06 ± 1.84^a^	–	–
*W*	54.06 ± 9.29^b^	12.87 ± 3.89^b^	33.82 ± 3.23^c^	64.89 ± 2.90^a^	−0.78 ± 0.70^b^	23.85 ± 1.08^b^	13.31 ± 8.26^a^	4.65 ± 0.91^a^
*H*	71.62 ± 6.36^c^	4.44 ± 4.76^c^	33.02 ± 7.40^c^	72.12 ± 3.19^b^	−1.90 ± 0.40^c^	34.68 ± 0.95^c^	31.72 ± 6.91^b^	15.70 ± 2.08^b^
*W/H*	62.03 ± 14.76^d^	10.71 ± 6.53^b^	29.13 ± 3.49^b^	69.97 ± 2.20^b^	1.86 ± 0.15^d^	21.66 ± 0.91^d^	20.96 ± 14.15^c^	6.02 ± 1.97^c^

*Note*: Δ*E* represents color differences. Different letters in a column represent statistically significant differences (*p*‐value <.05, Kruskal–Wallis, Games–Howell).

The color of the cakes depends on the Maillard reactions and caramelization during the baking process (crust color) and the ingredients used in the manufacture of the products (crumb color) (Majzoobi et al., 2014). As shown in Table 3, in the crumb, the formulation with *C. vulgaris* Honey resulted in brioches with higher color differences (Δ*E*) when compared with the control, reaching values of 15.70 ± 2.08. Similar average Δ*E* was obtained for the other incorporations, with values of 6.02 ± 1.97 and 4.65 ± 0.91 for the mixture of *C. vulgaris* White:Honey and for the *C. vulgaris* White, respectively. Nevertheless, statistically significant differences (*p*‐value <.05; Table 3) were found between these two Δ*E*. The higher Δ*E* of the mixture of *C. vulgaris* White:Honey when compared to the *C. vulgaris* White was mainly due to the L* values that showed that the samples were lighter than the control. No statistical differences were found in the values of L* when using *Chlorella vulgaris* White (*p*‐value >.05; Table 3). On the other hand, the a* and b* parameters were on average more alike to the control (Δ*a* = − 0.32 ± 0.15 and Δ*b* = − 0.40 ± 0.91) when using the microalgae mixture than the formulation with only *C. vulgaris* White (Δ*a* = − 2.96 ± 0.70 and Δ*b* = 1.79 ± 1.08).

Larger differences were verified between the control and the eggless brioche on crust browning. The control brioche presented a typical brown crust, while the eggless brioche crust did not brown as quickly during the allocated baking time (Figure 1). As previously mentioned, the crust color is a result of the reactions induced by the baking process, and the egg plays an important part on this front. Other studies regarding egg replacement in cakes have mentioned that higher protein ingredients originated in cakes with darker crusts (Hedayati et al., 2022). This is because of the Maillard reactions that occur in the presence of reducing sugars and proteins (Žilić et al., 2021). Darker crust (lower L* and highest a*) was verified in the control brioche than in the brioche with microalgae due to the latter's lower protein content caused by the absence of the egg. Similar results were obtained when using soy milk as egg substitute in cakes that was also justified by the difference in protein contents of the soy milk versus egg (Rahmati & Tehrani, 2014).

The main objective of this study was to use microalgae biomass to provide a coloration similar to the one provided by the eggs on bakery products. However, the effect of this replacement on the physical–chemical and nutritional properties was also analyzed. In this part, only one of the incorporations was further analyzed. In both cases, the use of *C. vulgaris* White and the mixture of *C. vulgaris* White:Honey demonstrated ability to provide color not very dissimilar to the one provided by the egg on the brioche. As previously mentioned, in terms of color, there was a Δ*E* of 6.02 ± 1.97 (when using the mixture), mainly due to the increased luminosity (L*) of the eggless brioche that denotes perceptible differences in color (Δ*E* >5) (Nunes, Fernandes, et al., 2020). Nevertheless, for the nutritional characterization of the eggless brioche, the formulation using a combination of the two *C. vulgaris* was selected due to the perceived nutritional benefits that both microalgae could provide to the product. *C. vulgaris* White presents higher contents of omega‐6 fatty acids, while *C. vulgaris* Honey presents higher contents of omega‐3 fatty acids. In addition, *C. vulgaris* Honey contains twice the amount of omega‐3 compared to *C. vulgaris* White, and it also contains lutein (Allma, 2021).

The comparison of the physical–chemical and proximate analysis of the control brioche and the eggless brioche is presented in Table 4.

**TABLE 4:** Physical–chemical and nutritional analysis of control brioche and eggless brioche with 1% *C. vulgaris* White and *C. vulgaris* Honey (1:1).

	Control brioche	Brioche with microalgae ([W/H] [1:1])
Weight loss (%)	8.43 ± 0.33^a^	6.20 ± 0.76^b^
pH		
Dough	5.67 ± 0.06^a^	5.26 ± 0.04^b^
Baked	5.52 ± 0.12^a^	5.21 ± 0.06^b^
*a*_w_	0.93 ± 0.00^a^	0.92 ± 0.01^b^
Texture		
Hardness (N)	2.62 ± 0.38^a^	3.12 ± 0.46^b^
Resilience (%)	10.85 ± 1.20^a^	9.85 ± 1.64^b^
Cohesion (%)	34.97 ± 2.75^a^	35.40 ± 2.49^a^
Springiness (%)	60.32 ± 4.74^a^	54.00 ± 4.95^b^
Color (Crumb)		
L*	64.13 ± 2.95^a^	69.97 ± 2.20^b^
a*	2.17 ± 0.39^a^	1.86 ± 0.15^b^
b*	22.06 ± 1.84^a^	21.66 ± 0.91^a^
ΔL*	–	5.84 ± 2.20
Δa*	–	−0.32 ± 0.15
Δb*	–	−0.40 ± 0.91
Δ*E*	–	6.02 ± 1.97
Moisture (%)	29.50 ± 0.31^a^	26.28 ± 0.52^b^
Ashes (%)	1.03 ± 0.03^a^	0.98 ± 0.02^b^
Protein (g/ 100 g DW)	11.48 ± 0.31^a^	9.02 ± 0.10^b^
Total fat (g/ 100 g DW)	16.05 ± 0.47^a^	14.86 ± 0.13^b^
Fatty acids (% of total fat)		
SFA	47^a^	48^b^
MUFA	39^a^	38^b^
PUFA	14^a^	14^b^
Carbohydrates (g/ 100 g DW)	38.50 ± 0.21^a^	43.88 ± 0.71^b^
Energy (Kcal/ 100 g)	335.54 ± 3.29^a^	344.87 ± 1.91^b^
TPC (mg GAE/g DW)	1.55 ± 0.35^a^	0.54 ± 0.06^b^
DPPH inhibition (μmol AAE/g DW)	0.80 ± 0.45^a^	1.18 ± 0.44^b^
FRAP (μmol AAE/g DW)	2.04 ± 0.52^a^	0.79 ± 0.30^b^

*Note*: Different letters in a row represent statistically significant differences (*t*‐test, *p*‐value <.05, Mann–Whitney U, *p*‐value <.05).

Eggs in bakery products are important for several technological characteristics, so their total substitution can lead to bakery products with different properties from the original egg product. The eggless brioches presented lower weight loss during the baking process, moisture, protein, total fat, and slightly lower pH. These results could have been caused by the absence of the egg and the fact that the egg was replaced by microalgae and water equivalent to egg white (approximately 60% of a whole egg) (Hedayati et al., 2022; Wilderjans et al., 2013). The lower moisture could have contributed to the differences in the texture of the brioches. Eggless brioche presented a significantly higher hardness and lower resilience and springiness (*p*‐value <.05; Table 4). Similar values to the control's cohesion were verified, although not statistically significant (*p*‐value >.05; Table 4).

The replacement of eggs in bakery has been attempted using various ingredients as substitutes (Hedayati et al., 2022; Yazici & Ozer, 2021). Lin et al. (2017) used soybean protein isolates (SPI) and various polysaccharides to replace the eggs in yellow cakes. The authors found that the replacement of the eggs increased the firmness and decreased the springiness of the product. The increase in the firmness was associated with the high‐water binding activity of the SPI that reduced the free water and amylose content, while the decrease in springiness was associated with the protein aggregation (Hedayati et al., 2022; Lin et al., 2017). In the eggless brioche produced in this work, slightly lower springiness values were observed, which may be the result of the lower protein content. The addition of higher amounts of microalgae could have contributed to mitigate the reduction in protein content since both *C. vulgaris* Honey and *C. vulgaris* White contain protein values around 30 g/100 g (Allma, 2021).

Additionally, the brioches were also evaluated for their total phenolic compounds and antioxidant potential by FRAP and DPPH assays. These different antioxidant quantification methods were used due to the distinct mechanism of action involved in the detected activity.

Low antioxidant activity was expected for these products due to the baking process, which can lead to the destruction of the compounds with antioxidant activity. In a study with cookies containing *C. vulgaris*, the authors observed a 50% reduction in the phenolic compounds during the baking process (Batista et al., 2017).

Both samples demonstrated low but quantifiable TPC, FRAP, and DPPH activities, with control brioche presenting significantly higher total phenolic compounds (1.55 ± 0.35 mg GAE/g) and ability to reduce the ferric ions (2.04 ± 0.52 μmol AAE/g) (*p*‐value <.05; Table 4). On the other hand, the eggless brioche demonstrated a significantly higher ability to reduce the DPPH radical (1.18 ± 0.44 μmol AAE/g) (*p*‐value <.05; Table 4).

The different growth conditions (autotrophic vs heterotrophic) and random mutagenesis that originates from the *C. vulgaris* biomass can result in the accumulation of lower bioactive compounds (Khemiri et al., 2022). The incorporation of 6% of different *C. vulgaris* (autotrophic [Organic *C. vulgaris*] and heterotrophic [*C. vulgaris* White, Honey, and Smooth]) in couscous resulted in distinct antioxidant potentials. Higher antioxidant activity was verified for the organic *C. vulgaris* with the highest TPC, FRAP, and DPPH activity. The application of heterotrophic *C. vulgaris* showed higher activity when using *C. vulgaris* Honey, presenting increases of 49%, 79%, and 41% in TPC, FRAP, and DPPH antioxidant activity, respectively, in comparison with the control. The incorporation of *C. vulgaris* White resulted in values similar to those of the control (Khemiri et al., 2022). On the other hand, snacks with 5% *C. vulgaris* Smooth presented a TPC of 1.10 mg GAE/g, a FRAP activity of 72.7 mg AAE/g DE, and a DPPH activity of 25.4 mg AAE/g DE, higher values than those of the control snack. Nevertheless, the antioxidant activity was inferior to that observed using the same percentage of *Spirulina*. These differences were related to the lower content of phytopigments present in the *C. vulgaris* Smooth caused by its growth conditions (heterotrophy) (Letras et al., 2022). In the present study, the microalga used was also grown under heterotrophic conditions, presenting even lower contents of phytopigments when compared to *C. vulgaris* Smooth. As a result, the reduced antioxidant activity when compared to the control can be attributed to this factor. Additionally, the low addition of microalgae (1%) may not have been enough to make up for the absence of eggs, considering that eggs themselves have been shown to possess antioxidant activity (Nimalaratne & Wu, 2015).

The microbial stability of the brioche samples (control and brioche with microalgae) was monitored during a 4‐day storage period (Table 5). According to the guidelines of the UK Health Protection Agency (2009), in general, the results of the microbiological analyses of the brioche presented satisfactory levels, with the exception of the total viable counts at 30°C that showed unsatisfactory values in the brioches with algae on days 1 and 4. Nevertheless, the values obtained are very close to the limit for being considered unsatisfactory (≥10^4^ CFU/g); yeasts probably contributed to these results as yeast counts at 25°C were higher in the brioches with algae. These differences could be due to the microalgae used, which also presented high values for total viable counts at 30°C (1.2 × 10^2^ cfu/g and <1.0 × 10^4^ cfu/g for *C. vulgaris* Honey and White, respectively) and yeasts at 25°C (<1.0 × 10^2^ cfu/g for both microalgae) (information taken from the microalgae datasheet). Possible contamination of the samples after baking could have also contributed to the obtained results. Nevertheless, it could be considered that both brioches, control and eggless brioche, were safe for consumption for at least three days after production.

**TABLE 5:** Microbial analysis of the brioche samples for the first 4 days after production.

Sample	Days	Microbial count at 30°C, (CFU/g)	Molds at 25°C, (CFU/g)	Yeast at 25°C, (CFU/g)	*Bacillus* spp., (CFU/g)	*B. Cereus*, (CFU/g)
Control	1	1.8 × 10^2^	<1.0 × 10^1^	1.2 × 10^2^	<1.0 × 10^1^	<1.0 × 10^1^
	2	8.5 × 10^2^	<1.0 × 10^1^	7.5 × 10^2^	Present but <4.0 × 10^1^	<1.0 × 10^1^
	3	4.7 × 10^2^	<1.0 × 10^1^	3.8 × 10^2^	Present but <4.0 × 10^1^	Present but <4.0 × 10^1^
	4	2.5 × 10^2^	EN = 7.0 × 10^1^	EN = 6.0 × 10^1^	<1.0 × 10^1^	<1.0 × 10^1^
Brioche W/H	1	5.1 × 10^4^	<1.0 × 10^1^	1.0 × 10^3^	Present but <4.0 × 10^1^	<1.0 × 10^1^
	2	9.5 × 10^3^	<1.0 × 10^1^	3.5 × 10^3^	Present but <4.0 × 10^1^	<1.0 × 10^1^
	3	4.3 × 10^3^	<1.0 × 10^1^	4.0 × 10^3^	2.3 × 10^2^	<1.0 × 10^1^
	4	1.1 × 10^4^	<1.0 × 10^1^	6.6 × 10^3^	<1.0 × 10^1^	<1.0 × 10^1^

Abbreviation: EN, estimated number.

**FIGURE 2:**
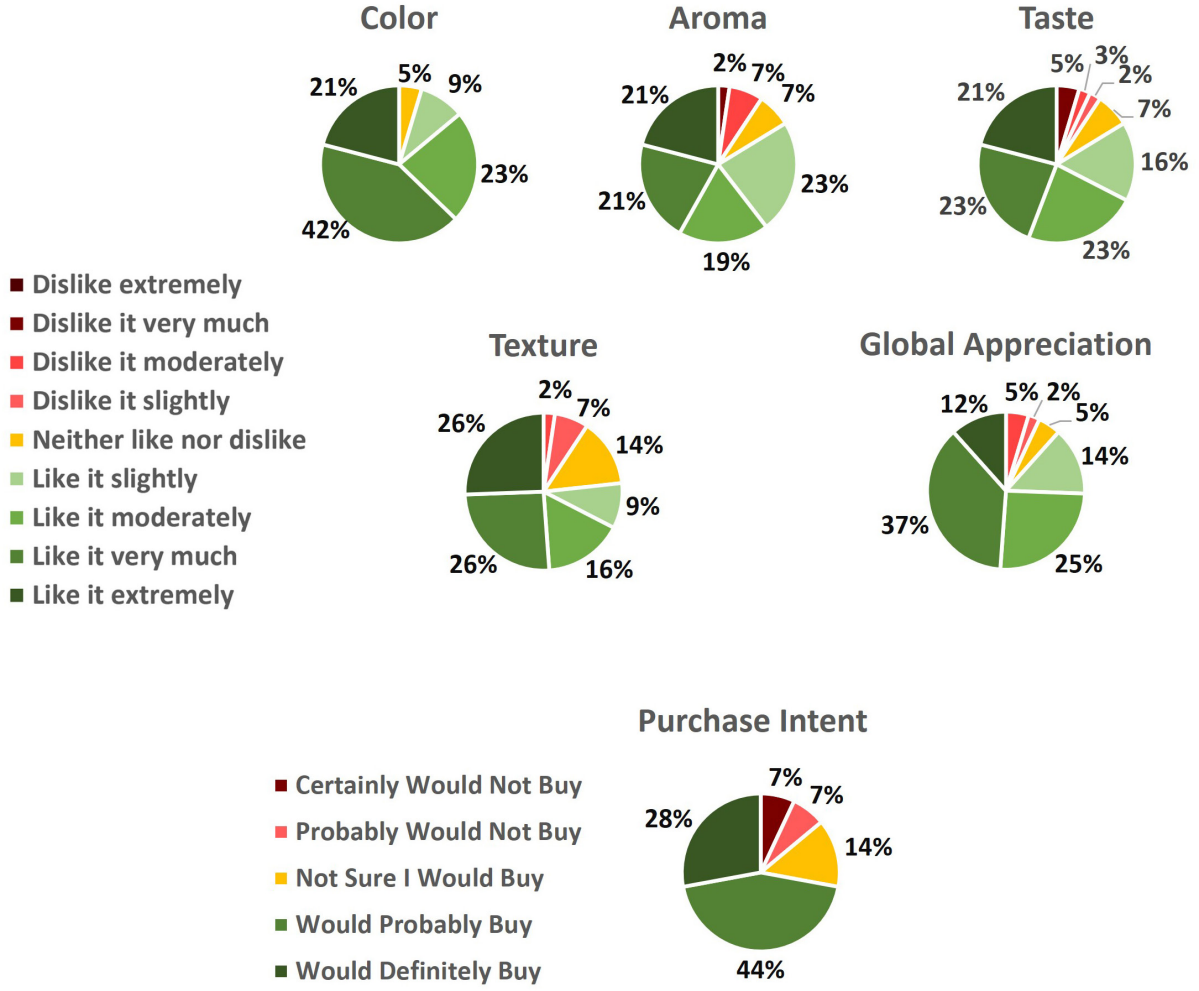
Sensory results and purchase intent of the eggless brioche.

As a way to evaluate the market potential of the eggless brioche, sensory evaluation was performed. Color, aroma, taste, texture, and global appreciation of the brioches were evaluated, and the purchase intent was assessed (Figure 2).

Overall, the eggless brioche with microalgae obtained positive responses in all parameters evaluated. Responses were considered positive from “like it slightly” to “like it extremely,” which accounted for 95% of the responses in color, 84% in aroma and taste, 77% in texture, and 88% in the global appreciation. As for the purchase intent, 72% of the consumers indicated that they would “probably buy” and “definitely buy.” Nevertheless, this is only a demonstration of the potential of the product since a larger sample size (>80) is needed to yield stronger results (Drake et al., 2023).

As previously mentioned, the main objective of the present study was to replace the eggs and provide color using *C. vulgaris*. Nevertheless, the results suggest that the use of microalgae has the potential to being used not only to provide color but also to create eggless versions of brioche with good sensory acceptability.

### 3.2 Fondant

Using the whole microalgae as a fondant colorant was challenging due to the intense flavor provided by the microalgae tested (results not shown). Therefore, microalgae extracts were used to color the fondant. Although it is reported that chlorophyll is one of the main contributors to the algae flavor (Schüler et al., 2020), by extracting these compounds it becomes possible to introduce less amounts of the extract to achieve an appealing color with the reduced flavor intensity.

*T. chuii* extracts were produced using ethanol 80% v/v at a proportion of 1:20 (biomass: solvent) (Kulkarni & Nikolov, 2018). Two consecutive extractions ensured that the maximum pigment extraction was achieved. The concentration of the different pigments in the *T. chuii* extracts, the extraction yield, and the antioxidant capacity of the extracts are presented in Table 6.

**TABLE 6:** Total concentration of pigments (chlorophyll a, chlorophyll b, and carotenoids), extraction yield, total phenolic compounds, and DPPH and FRAP activities of the extracts from *Tetraselmis chuii*.

	Total (mg/g)	Extraction yield (%)	TPC (mg GAE/g)	DPPH inhibition (μmol AAE/ g)	FRAP (μmol AAE/ g)
Chlorophyll a	2.23 ± 0.10	33.34 ± 0.52	6.30 ± 1.96	12.22 ± 4.78	24.04 ± 6.06
Chlorophyll b	2.45 ± 0.08				
Carotenoids	0.04 ± 0.00				

The extracts obtained in this study exhibited similar concentrations of chlorophyll *b* (2.45 ± 0.08 mg/g) and chlorophyll *a* (2.23 ± 0.10 mg/g) along with trace amounts of carotenoids (0.04 ± 0.00 mg/g). Higher contents of carotenoids than the ones obtained in this study have been previously reported, with some studies indicating maximum values of 8.5 mg/g for *T. chuii* in methanolic extracts, while lower values were observed for other species of *Tetraselmis* (Banskota et al., 2019; Goiris et al., 2012; Paterson et al., 2023). The variations in carotenoid amounts can be associated with the different biomass growth conditions and environmental stress factors, which have been found to influence the production of pigments (Goiris et al., 2012; Paterson et al., 2023).

The extracts from *T. chuii* also exhibited antioxidant activity, as shown in Table 6. Previous studies have reported varying ranges of antioxidant activity for *T. chuii* extracts, depending on the types of solvents used in the extraction and the concentration used on the assays. Similar total phenolic contents have been previously determined for water/ethanol extracts from *Tetraselmis* spp., presenting values of 3.74 ± 0.10 mg GAE/g DW (Goiris et al., 2012). Higher values were obtained for *T. chuii* using other extraction solvents (non‐food grade). The total phenolic amount in *T. chuii* was quantified in the order of 20 mg GAE/g DW in acetone/hydrochloric acid extracts, while lower values were obtained in ethanol (13.61 mg GAE/g), methanol (8.6 mg GAE/g DW), and water and DMSO extracts (values ranging from 4.38 to 6.70 mg GAE/g DW) (Custódio et al., 2012; Gangadhar et al., 2016; Kokkali et al., 2020; Tibbetts et al., 2015).

At a concentration of 0.1 g/mL, a DPPH scavenging activity of 17.40 ± 3.33% (equivalent to 12.22 ± 4.78 μmol AAE/g [Table 6]) was obtained. Higher DPPH scavenging activity was observed for methanol and hexane extracts with values of 45.8 ± 2.5 and 68.1 ± 2.3%, respectively, at a concentration of 10 mg/mL (Custódio et al., 2012). At a concentration of 200 μg/mL, methanolic extracts presented 45% of DPPH scavenging activity (Banskota et al., 2019), while ethanolic extracts presented around 67% DPPH scavenging activity (IC_50_ of 1.14 ± 0.01 mg/mL) (Silva et al., 2022). Lipidic extracts from *T. chuii* demonstrated in the DPPH essay an IC_20_ of 225.7 ± 6.9 μg/mL (Conde et al., 2021).

As for the FRAP activity, other studies using *T. chuii* extracts also reported FRAP activity. Custódio et al. (2012) reported in this microalga higher ability to chelate Fe^2+^ when using hexane (77.8 ± 6.2% at 1 mg/mL) than methanol (32.2 ± 5.0% at 10 mg/mL). In *T. chuii* ethanolic extracts, the ability to chelate Fe^2+^ was 16.6 ± 0.7% when using a concentration of 1 mg/mL and 88.9 ± 2.7% at a concentration of 10 mg/mL (Gangadhar et al., 2016; Silva et al., 2022). Hydroethanolic *Tetraselmis* spp. extracts (100 mg/mL concentration) presented FRAP results of 46.58 ± 0.60 μmol Trolox equivalent/g DW (Goiris et al., 2012).

Various factors can contribute to different pigment contents and bioactivities. The growth conditions and biomass processing can contribute to the potential increase or decrease in pigments and compounds with antioxidant activity (Goiris et al., 2012). On the other hand, the extraction solvent used influences the polarity of the compounds extracted, with polar solvents (ethanol and methanol) extracting more phenolic compounds and some PUFA, while non‐polar solvents (hexane) extract more pigments, polyphenols, and PUFA (Custódio et al., 2012; Silva et al., 2022). Water is only able to extract polar compounds such as polyphenols and polysaccharides (Silva et al., 2022). The application in food products can hinder the use of some of the more efficient solvents for antioxidant compounds due to their non‐food grade nature.

**FIGURE 3:**
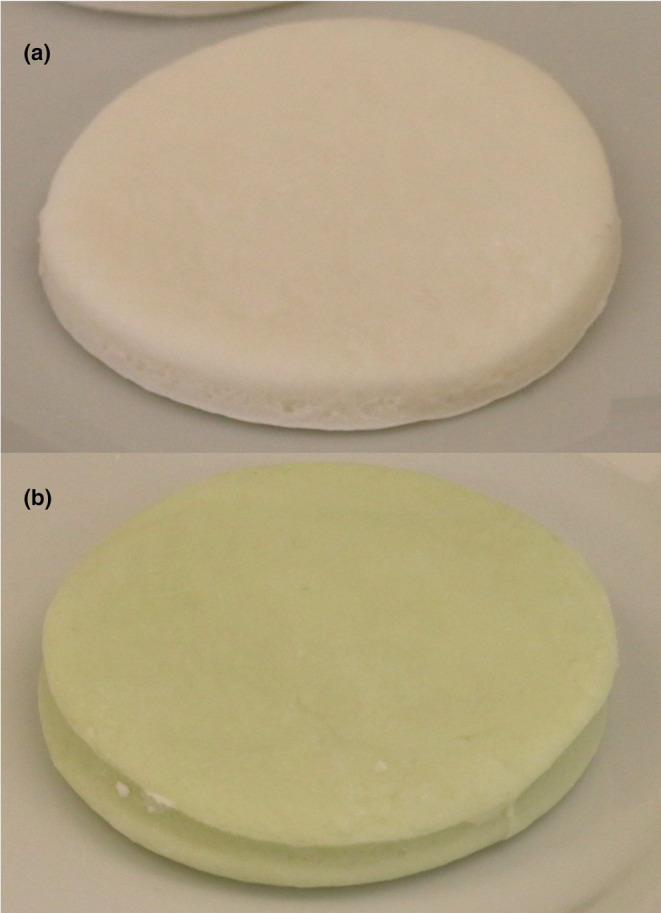
Representation of the control fondant (a) and the fondant with 0.05% of *T. chuii* extract (b).

The potential of using the *T. chuii* extracts in food as colorants was evaluated by applying the extracts to fondant. This product was chosen due to the fact that it is available in a wide array of colors that are often from non‐natural sources. The level of extract addition to the fondant was selected through sensory testing, and it was determined that the maximum percentage of extract that achieved acceptable sensory characteristics was 0.05% (w/w). Figure 3 shows samples of fondant with and without the incorporation of extract.

The effect of the incorporation of *T. chuii* extract in the fondant was evaluated by comparing the physical–chemical and proximate nutritional analysis of the fondant with and without extract (Table 7).

**TABLE 7:** Physical–chemical and nutritional analysis of the fondants with and without 0.05% *T. chuii* extract.

	Control fondant	Fondant (*T. Chuii* extract)
pH	4.53 ± 0.02^a^	4.31 ± 0.03^b^
*a*_w_	0.85 ± 0.00^a^	0.85 ± 0.00^b^
Texture		
Hardness (N)	0.33 ± 0.03^a^	0.33 ± 0.03^a^
Color		
L*	90.17 ± 1.07^a^	83.76 ± 1.18^b^
a*	3.58 ± 0.09^a^	−4.68 ± 0.71^b^
b*	4.54 ± 0.47^a^	15.05 ± 1.15^b^
ΔL*	–	−6.41 ± 1.18
Δa*	–	−8.26 ± 0.71
Δb*	–	10.51 ± 1.15
Δ*E*	–	14.86 ± 1.41
Moisture (%)	16.15 ± 0.36^a^	15.88 ± 0.36^b^
Ashes (%)	0.05 ± 0.01^a^	0.06 ± 0.01^b^
Protein (g/100 g)	1.73 ± 0.06^a^	1.83 ± 0.06^a^
Total Fat (g/100 g)	0.28 ± 0.07^a^	0.54 ± 0.07^b^
Carbohydrates (g/100 g)	81.79 ± 0.19^a^	81.68 ± 0.17^a^
Energy (Kcal/100 g)	336.56 ± 1.22^a^	338.98 ± 1.30^a^

*Note*: Different letters in a row represent statistically significant differences (*t*‐test, *p*‐value <.05).

Both fondants presented similar average results for *a*_w_, texture, moisture, and ashes; nevertheless, statistical differences were identified between the samples (*p*‐value .05; Table 7). The extract also provided a fondant with slightly lower pH, and higher fat content (*p*‐value .05; Table 7). The *T. chuii* extract caused a noticeable increase in fat content when compared with the control reaching values of 0.54 ± 0.07 g of fat/ 100 g (fresh weight) versus 0.28 ± 0.07 g fat/ 100 g (fresh weight) for the extract and control fondant, respectively (*p*‐value <.05; Table 7). This may be caused by the simultaneous extraction of fat along with the pigment extraction, resulting in an augmented fat content in the fondant where the extract was used. In terms of hardness, protein, carbohydrates, and energy, no differences were found (*p*‐value >.05; Table 7).

In terms of color, naturally the extract decreased the L* parameter while increasing the values of a* and b*, providing a green coloration to the product. *T. chuii* extracts demonstrated the potential of being used as natural colorants, with the ability to retain their color over time. After a period of storage at 4°C, a color analysis showed that some color was retained presenting a color difference (Δ*E*) of only 5.60 ± 1.40 between the fondant with extract at time 0 and after 4 months of storage. This difference was mainly due to difference in luminosity (less luminosity) and the green coordinates (less green) (*p*‐value .05; Table 7). This was expected since in previous studies, the reduction of the green coordinate was used as a way to evaluate chlorophyll degradation (Ferreira et al., 2023; Shen et al., 2010). Chlorophylls have been shown to be sensitive to various conditions; nevertheless, ethanolic extracts from *Chlorella vulgaris* showed good stability at <28°C in the dark, with their incorporation in rice being visually stable for 3 days (4°C) (Ferreira et al., 2023).

Finally, the microbial stability of the fondant was evaluated during a storage period of 28 days at room temperature, evaluating total viable counts at 30°C, molds and yeast at 25°C, *Bacillus* spp. and *Bacillus cereus* (Table 8).

**TABLE 8:** Microbial analysis of the fondant samples for a period of 28 days.

Sample	Days	Microbial count at 30°C, (ufc/g)	Molds at 25°C, (ufc/g)	Yeast at 25°C, (ufc/g)	*Bacillus* spp., (ufc/g)	*B. Cereus*, (ufc/g)
Control fondant	1	EN = 8.0 × 10^1^	<1.0 × 10^1^	1.0 × 10^2^	<1.0 × 10^1^	1.7 × 10^2^
	7	EN = 6.0 × 10^1^	<1.0 × 10^1^	1.1 × 10^2^	<1.0 × 10^1^	1.4 × 10^2^
	14	2.1 × 10^3^	<1.0 × 10^1^	8.0 × 10^3^	<1.0 × 10^1^	<1.0 × 10^1^
	21	4.2 × 10^3^	<1.0 × 10^1^	6.1 × 10^2^	<1.0 × 10^1^	<1.0 × 10^1^
	28	EN = 4.0 × 10^1^	<1.0 × 10^1^	2.5 × 10^2^	<1.0 × 10^1^	<1.0 × 10^1^
Fondant with *T. chuii* Extract	1	2.4 × 10^2^	<1.0 × 10^1^	1.3 × 10^2^	<1.0 × 10^1^	6.7 × 10^2^
	7	1.2 × 10^2^	<1.0 × 10^1^	1.1 × 10^2^	<1.0 × 10^1^	1.8 × 10^2^
	14	5.2 × 10^2^	<1.0 × 10^1^	6.6 × 10^2^	<1.0 × 10^1^	<1.0 × 10^1^
	21	7.1 × 10^2^	<1.0 × 10^1^	1.6 × 10^3^	<1.0 × 10^1^	<1.0 × 10^1^
	28	Present but <4.0 × 10^1^	Present but <4.0 × 10^1^	2.6 × 10^3^	<1.0 × 10^1^	<1.0 × 10^1^

Abbreviation: EN, estimated number.

As can be confirmed, all the values remained within acceptable levels throughout the monitored period, indicating that the fondants were microbiologically stable for at least 28 days at room temperature.

## 4 CONCLUSION

The potential of using microalgae, both whole biomass and extracts, as alternative for synthetic colorants was evaluated on two pastry products (brioche‐type bread and fondant).

In brioche‐type bread, 1% *C. vulgaris* (White: Honey [1:1]) biomass showed the ability to replace the egg with minimal disturbances in the physical properties of the product. The nutritional aspect of the brioche presented some differences, with the eggless brioche having lower fat and protein content. Further studies should be made by increasing the amount of microalgae used to try to increase the proteins without increasing the total fat. A preliminary consumer acceptance test showed that the brioche had good acceptance, demonstrating the possible use of *C. vulgaris* (White and Honey) as egg substitute to provide color in bakery products.

Hydroethanolic *T. chuii* extracts were successfully used as colorant in fondant providing a green color at a percentage as low as 0.05%, with minimal algae taste. The extracts presented considerable amounts of total phenolic compounds and antioxidant activity. The addition of the extracts to the fondant did not influence the physical–chemical and nutritional characteristics of the fondant being able to retain the color for at least 4 months.

This study has shown that microalgae have good potential to be used as natural colorant in confectionary and pastry products, having the advantage of nutritionally enriching them.

## AUTHOR CONTRIBUTIONS

**Tatiana Pereira:** Investigation (lead); visualization (equal); writing – original draft (equal). **Sónia Barroso:** Supervision (equal); validation (equal); writing – review and editing (equal). **Filipa R. Pinto:** Writing – review and editing (equal). **Frederica Silva:** Investigation (supporting); writing – review and editing (supporting). **Paula Teixeira:** Investigation (equal); writing – review and editing (equal). **Susana Mendes:** Formal analysis (equal); writing – review and editing (equal). **Maria M. Gil:** Conceptualization (equal); funding acquisition (equal); project administration (equal); resources (equal); writing – review and editing (equal).

## FUNDING INFORMATION

This work was funded by national funds through FCT – Fundação para a Ciência e a Tecnologia, I.P., under the project MARE (UIDB/04292/2020 and UIDP/04292/2020), the project LA/P/0069/2020 granted to the Associate Laboratory ARNET, the grants awarded to Tatiana Pereira (2021.07791.BD) and Frederica Silva (UI/BD/151098/2021), the project ALGAVALOR – Microalgas: Integrated production and valorization of biomass and its various applications (POCI‐01‐0247‐FEDER‐035234, LISBOA‐01‐0247‐FEDER‐035234, ALG‐01‐0247‐FEDER‐035234), and the project PRIMA/0002/2022‐MoreMedDiet: More on the adoption of a healthy Mediterranean Diet, within the Partnership for Research and Innovation in the Mediterranean Area (PRIMA).

## CONFLICT OF INTEREST STATEMENT

The authors declare that they do not have any conflict of interest.

## ETHICS STATEMENT

Informed Consent: Participants gave verbal consent for the sensory evaluation, and they were able to withdraw from the survey at any time without giving a reason. The products tested were safe for consumption.

## Data Availability

The data that support the findings of this study are available on request from the corresponding author.

## References

[fsn34394-bib-0001] Allma . (2021). Allma . https://www.allmashop.com/index.php

[fsn34394-bib-0002] Amchova, P. , Kotolova, H. , & Ruda‐Kucerova, J. (2015). Health safety issues of synthetic food colorants. Regulatory Toxicology and Pharmacology, 73(3), 914–922. 10.1016/j.yrtph.2015.09.026 26404013

[fsn34394-bib-0003] AOAC 992.23‐1992 . (1998). Crude protein in cereal grains and oilseeds. Generic Combustion Method. AOAC International, Rockville, ML, USA.

[fsn34394-bib-0004] Banskota, A. H. , Sperker, S. , Stefanova, R. , McGinn, P. J. , & O'Leary, S. J. B. (2019). Antioxidant properties and lipid composition of selected microalgae. Journal of Applied Phycology, 31(1), 309–318. 10.1007/s10811-018-1523-1

[fsn34394-bib-0005] Batista, A. P. , Niccolai, A. , Fradinho, P. , Fragoso, S. , Bursic, I. , Rodolfi, L. , Biondi, N. , Tredici, M. R. , Sousa, I. , & Raymundo, A. (2017). Microalgae biomass as an alternative ingredient in cookies: Sensory, physical and chemical properties, antioxidant activity and in vitro digestibility. Algal Research, 26(June), 161–171. 10.1016/j.algal.2017.07.017

[fsn34394-bib-0006] Benzie, I. F. F. , & Strain, J. J. (1996). The ferric reducing ability of plasma (FRAP) as a measure of “antioxidant power”: The FRAP assay. Analytical Biochemistry, 239(1), 70–76. 10.1006/abio.1996.0292 8660627

[fsn34394-bib-0007] Brand‐Williams, W. , Cuvelier, M. E. , & Berset, C. (1995). Use of a free radical method to evaluate antioxidant activity. LWT ‐ Food Science and Technology, 28(1), 25–30. 10.1016/S0023-6438(95)80008-5

[fsn34394-bib-0008] Conde, T. A. , Neves, B. F. , Couto, D. , Melo, T. , Neves, B. , Costa, M. , Silva, J. , Domingues, P. , & Domingues, M. R. (2021). Microalgae as sustainable bio‐factories of healthy lipids: Evaluating fatty acid content and antioxidant activity. Marine Drugs, 19(7), 357. 10.3390/md19070357 34201621 PMC8307217

[fsn34394-bib-0009] Correia, P. M. R. , Gonzaga, M. , Batista, L. M. , Beirão‐costa, L. , & Guiné, R. F. P. (2015). Development and characterization of wheat bread with lupin flour. International Scholarly and Scientific Research & Innovation, 9(10), 923–927. 10.5281/zenodo.1109123

[fsn34394-bib-0010] Custódio, L. , Justo, T. , Silvestre, L. , Barradas, A. , Duarte, C. V. , Pereira, H. , Barreira, L. , Rauter, A. P. , Alberício, F. , & Varela, J. (2012). Microalgae of different phyla display antioxidant, metal chelating and acetylcholinesterase inhibitory activities. Food Chemistry, 131(1), 134–140. 10.1016/j.foodchem.2011.08.047

[fsn34394-bib-0011] Dey, S. , & Nagababu, B. H. (2022). Applications of food color and bio‐preservatives in the food and its effect on the human health. Food Chemistry Advances, 1, 100019. 10.1016/j.focha.2022.100019

[fsn34394-bib-0012] Drake, M. A. , Watson, M. E. , & Liu, Y. (2023). Sensory analysis and consumer preference: Best practices. Annual Review of Food Science and Technology, 14(1), 427–448. 10.1146/annurev-food-060721-023619 36972161

[fsn34394-bib-0013] Fernández, A. , Grienke, U. , Soler‐Vila, A. , Guihéneuf, F. , Stengel, D. B. , & Tasdemir, D. (2015). Seasonal and geographical variations in the biochemical composition of the blue mussel (*Mytilus edulis* L.) from Ireland. Food Chemistry, 177, 43–52. 10.1016/j.foodchem.2014.12.062 25660856

[fsn34394-bib-0014] Ferreira, A. S. , Pereira, L. , Canfora, F. , Silva, T. H. , Coimbra, M. A. , & Nunes, C. (2023). Stabilization of natural pigments in ethanolic solutions for food applications: The case study of *Chlorella vulgaris* . Molecules, 28(1), 408. 10.3390/molecules28010408 36615600 PMC9822436

[fsn34394-bib-0015] Folch, J. , Lees, M. , & Sloane Stanley, G. H. (1957). A simple method for the isolation and purification of total lipides from animal tissues. The Journal of Biological Chemistry, 226(1), 497–509. 10.1016/s0021-9258(18)64849-5 13428781

[fsn34394-bib-0016] Gangadhar, K. N. , Pereira, H. , Rodrigues, M. J. , Custódio, L. , Barreira, L. , Malcata, F. X. , & Varela, J. (2016). Microalgae‐based unsaponifiable matter as source of natural antioxidants and metal chelators to enhance the value of wet *Tetraselmis chuii* biomass. Open Chemistry, 14(1), 299–307. 10.1515/chem-2016-0029

[fsn34394-bib-0017] Ghendov‐Mosanu, A. , Ungureanu‐Iuga, M. , Mironeasa, S. , & Sturza, R. (2022). *Aronia* extracts in the production of confectionery masses. Applied Sciences, 12(15), 7664. 10.3390/app12157664

[fsn34394-bib-0018] Goiris, K. , Muylaert, K. , Fraeye, I. , Foubert, I. , De Brabanter, J. , & De Cooman, L. (2012). Antioxidant potential of microalgae in relation to their phenolic and carotenoid content. Journal of Applied Phycology, 24(6), 1477–1486. 10.1007/s10811-012-9804-6

[fsn34394-bib-0019] Grand View Research . (2024). Food Colors Market Size, Share & Trends Analysis Report By Type (Natural, Synthetic, Nature‐identical), By Form (Powder, Liquid, Gel & Paste), By Source (Plants, Animals, & Insects, Microorganisms, Petroleum), By Application, By Region, And Segment Forecasts, 2024–2030 . https://www.grandviewresearch.com/industry‐analysis/food‐colorants‐market

[fsn34394-bib-0500] Health Protection Agency . (2009). Guidelines for Assessing the Microbiological Safety of Ready‐to‐Eat Foods. London: Health Protection Agency.

[fsn34394-bib-0021] Hedayati, S. , Jafari, S. M. , Babajafari, S. , Niakousari, M. , & Mazloomi, S. M. (2022). Different food hydrocolloids and biopolymers as egg replacers: A review of their influences on the batter and cake quality. Food Hydrocolloids, 128, 107611. 10.1016/j.foodhyd.2022.107611

[fsn34394-bib-0022] ISO 16634 . (2005). Determination of the total nitrogen content by combustion according to the Dumas principle and calculation of the crude protein content of cereals, pulses, milled cereal products, oilseeds and animal feeding stuffs.

[fsn34394-bib-0023] ISO 4833‐1 . (2013). Microbiology of the food chain — Horizontal method for the enumeration of microorganisms — Part 1: Colony count at 30°C by the pour plate technique .

[fsn34394-bib-0024] ISO 7932 . (2004). Microbiology of food and animal feeding stuffs — Horizontal method for the enumeration of presumptive Bacillus cereus — Colony‐count technique at 30°C.

[fsn34394-bib-0025] Khemiri, S. , Khelifi, N. , Nunes, M. C. , Ferreira, A. , Gouveia, L. , Smaali, I. , & Raymundo, A. (2020). Microalgae biomass as an additional ingredient of gluten‐free bread: Dough rheology, texture quality and nutritional properties. Algal Research, 50, 101998. 10.1016/j.algal.2020.101998

[fsn34394-bib-0026] Khemiri, S. , Nunes, M. C. , Raymundo, A. , & Smaali, I. (2022). *In vitro* starch digestibility and estimation of glycemic index in algae‐based couscous. International Journal of Food Science and Technology, 57(11), 7245–7253. 10.1111/ijfs.16073

[fsn34394-bib-0027] Kokkali, M. , Martí‐Quijal, F. J. , Taroncher, M. , Ruiz, M.‐J. , Kousoulaki, K. , & Barba, F. J. (2020). Improved extraction efficiency of antioxidant bioactive compounds from *Tetraselmis chuii* and *Phaedoactylum tricornutum* using pulsed electric fields. Molecules, 25(17), 3921. 10.3390/molecules25173921 32867350 PMC7504414

[fsn34394-bib-0028] Kulkarni, S. , & Nikolov, Z. (2018). Process for selective extraction of pigments and functional proteins from *Chlorella vulgaris* . Algal Research, 35, 185–193. 10.1016/j.algal.2018.08.024

[fsn34394-bib-0029] Letras, P. , Oliveira, S. , Varela, J. , Nunes, M. C. , & Raymundo, A. (2022). 3D printed gluten‐free cereal snack with incorporation of *spirulina* (*Arthrospira platensis*) and/or *Chlorella vulgaris* . Algal Research, 68, 102863. 10.1016/j.algal.2022.102863

[fsn34394-bib-0030] Lin, M. , Hong, S. , Yang, H. , Yang, B. , & Li, H. (2017). Replacement of eggs with soybean protein isolates and polysaccharides to prepare yellow cakes suitable for vegetarians. Food Chemistry, 229, 663–673. 10.1016/j.foodchem.2017.02.132 28372229

[fsn34394-bib-0031] Luzardo‐Ocampo, I. , Ramírez‐Jiménez, A. K. , Yañez, J. , Mojica, L. , & Luna‐Vital, D. a. (2021). Technological applications of natural colorants in food systems: A review. Food, 10(3), 1–34. 10.3390/foods10030634 PMC8002548 33802794

[fsn34394-bib-0032] Maehle, N. , & Skjeret, F. (2022). Microalgae‐based food: Purchase intentions and willingness to pay. Future Foods, 6, 100205. 10.1016/j.fufo.2022.100205

[fsn34394-bib-0033] Majzoobi, M. , Ghiasi, F. , Habibi, M. , Hedayati, S. , & Farahnaky, A. (2014). Influence of soy protein isolate on the quality of batter and sponge cake. Journal of Food Processing and Preservation, 38(3), 1164–1170. 10.1111/jfpp.12076

[fsn34394-bib-0034] Market Data Forecast . (2024). Global Egg Replacers Market Size, Share, Trends, COVID‐19 Impact & Growth Forecast Report – Segmented By Form (Dry And Liquid), Source (Plant, Animal), Ingredient (Dairy Proteins, Starch, Algal Flour, Soy‐Based Products), Application (Bakery & Confectionery, Savories, Sauces, Dressings & Spreads) And Region (North America, Europe, APAC, Latin America, Middle East And Africa) – Industry Analysis From 2024 To 2029 . https://www.marketdataforecast.com/market‐reports/egg‐replacers‐market

[fsn34394-bib-0035] Ngamwonglumlert, L. , Devahastin, S. , & Chiewchan, N. (2017). Natural colorants: Pigment stability and extraction yield enhancement via utilization of appropriate pretreatment and extraction methods. Critical Reviews in Food Science and Nutrition, 57(15), 3243–3259. 10.1080/10408398.2015.1109498 26517806

[fsn34394-bib-0036] Nimalaratne, C. , & Wu, J. (2015). Hen egg as an antioxidant food commodity: A review. Nutrients, 7(10), 8274–8293. 10.3390/nu7105394 26404361 PMC4632414

[fsn34394-bib-0037] NP 2032 . (2009). Produtos da pesca e da aquicultura. Determinação do teor de cinzas. Instituto Português da Qualidade.

[fsn34394-bib-0038] NP 3277‐1 . (1987). Microbiologia alimentar. Contagem de bolores e leveduras. Parte 1: Incubação a 25°C. Instituto Português da Qualidade.

[fsn34394-bib-0039] Nunes, M. C. , Fernandes, I. , Vasco, I. , Sousa, I. , & Raymundo, A. (2020). *Tetraselmis chuii* as a sustainable and healthy ingredient to produce gluten‐free bread: Impact on structure, colour and bioactivity. Food, 9(5), 579. 10.3390/foods9050579 PMC7278787 32375425

[fsn34394-bib-0040] Nunes, M. C. , Graça, C. , Vlaisavljević, S. , Tenreiro, A. , Sousa, I. , & Raymundo, A. (2020). Microalgal cell disruption: Effect on the bioactivity and rheology of wheat bread. Algal Research, 45, 101749. 10.1016/j.algal.2019.101749

[fsn34394-bib-0041] Paterson, S. , Gómez‐Cortés, P. , de la Fuente, M. A. , & Hernández‐Ledesma, B. (2023). Bioactivity and digestibility of microalgae *Tetraselmis* sp. and *Nannochloropsis* sp. As basis of their potential as novel functional foods. Nutrients, 15(2), 477. 10.3390/nu15020477 36678348 PMC9861193

[fsn34394-bib-0042] Pereira, T. , Costa, S. , Barroso, S. , Teixeira, P. , Mendes, S. , & Gil, M. M. (2024). Development and optimization of high‐protein and low‐saturated fat bread formulations enriched with lupin and microalgae. LWT, 191, 115612. 10.1016/j.lwt.2023.115612

[fsn34394-bib-0043] Peris, M. , Rubio‐Arraez, S. , Castelló, M. L. , & Ortolá, M. D. (2019). From the laboratory to the kitchen: New alternatives to healthier bakery products. Food, 8(12), 1–27. 10.3390/foods8120660 PMC6963723 31835412

[fsn34394-bib-0044] Rahmati, N. F. , & Tehrani, M. M. (2014). Influence of different emulsifiers on characteristics of eggless cake containing soy milk: Modeling of physical and sensory properties by mixture experimental design. Journal of Food Science and Technology, 51(9), 1697–1710. 10.1007/s13197-013-1253-y 25190826 PMC4152492

[fsn34394-bib-0045] Regulation (EU) 2017/2470 . (2017). Commission implementing regulation (EU) 2017/2470 of 20 December 2017 establishing the union list of novel foods in accordance with regulation (EU) 2015/2283 of the European Parliament and of the council on novel foods. Official Journal of the European Union, 351(258), 72–201. http://eur‐lex.europa.eu/legal‐content/EN/TXT/PDF/?uri=CELEX:32017R2470&from=EN

[fsn34394-bib-0046] Schüler, L. , Greque de Morais, E. , Trovão, M. , Machado, A. , Carvalho, B. , Carneiro, M. , Maia, I. , Soares, M. , Duarte, P. , Barros, A. , Pereira, H. , Silva, J. , & Varela, J. (2020). Isolation and characterization of novel *Chlorella vulgaris* mutants with low chlorophyll and improved protein contents for food applications. Frontiers in Bioengineering and Biotechnology, 8(May), 1–10. 10.3389/fbioe.2020.00469 32509750 PMC7248561

[fsn34394-bib-0047] Shen, S. C. , Hsu, H. Y. , Huang, C. N. , & Wu, J. S. B. (2010). Color loss in ethanolic solutions of chlorophyll a. Journal of Agricultural and Food Chemistry, 58(13), 8056–8060. 10.1021/jf101214g 20521818

[fsn34394-bib-0048] Sigurdson, G. T. , Tang, P. , & Giusti, M. M. (2017). Natural colorants: Food colorants from natural sources. Annual Review of Food Science and Technology, 8(12), 1–20. 10.1146/annurev-food-030216-025923 28125346

[fsn34394-bib-0049] Silva, M. , Kamberovic, F. , Uota, S. T. , Kovan, I. M. , Viegas, C. S. B. , Simes, D. C. , Gangadhar, K. N. , Varela, J. , & Barreira, L. (2022). Microalgae as potential sources of bioactive compounds for functional foods and pharmaceuticals. Applied Sciences (Switzerland), 12(12), 5877. 10.3390/app12125877

[fsn34394-bib-0050] Singleton, V. L. , & Rossi, J. A. (1965). Colorimetry of total phenolics with phosphomolybdic‐phosphotungstic acid reagents. The American Journal of Enology and Viticulture, 16, 144–158.

[fsn34394-bib-0051] Spence, C. (2015). On the psychological impact of food colour. Flavour, 4(1), 1–16. 10.1186/s13411-015-0031-3

[fsn34394-bib-0052] Tibbetts, S. M. , Milley, J. E. , & Lall, S. P. (2015). Chemical composition and nutritional properties of freshwater and marine microalgal biomass cultured in photobioreactors. Journal of Applied Phycology, 27(3), 1109–1119. 10.1007/s10811-014-0428-x

[fsn34394-bib-0053] van Durme, J. , Goiris, K. , de Winne, A. , de Cooman, L. , & Muylaert, K. (2013). Evaluation of the volatile composition and sensory properties of five species of microalgae. Journal of Agricultural and Food Chemistry, 61(46), 10881–10890. 10.1021/jf403112k 24138670

[fsn34394-bib-0054] Waterhouse, A. L. (2003). Determination of total phenolics. In Current protocols in food analytical chemistry. John Wiley & Sons, Inc. 10.1002/0471142913.fai0101s06

[fsn34394-bib-0055] Wilderjans, E. , Luyts, A. , Brijs, K. , & Delcour, J. A. (2013). Ingredient functionality in batter type cake making. Trends in Food Science and Technology, 30(1), 6–15. 10.1016/j.tifs.2013.01.001

[fsn34394-bib-0056] Yazici, G. N. , & Ozer, M. S. (2021). A review of egg replacement in cake production: Effects on batter and cake properties. Trends in Food Science & Technology, 111, 346–359. 10.1016/j.tifs.2021.02.071

[fsn34394-bib-0057] Zar, J. H. (2010). Biostatistical analysis (5th ed.). Prentice Hall.

[fsn34394-bib-0058] Žilić, S. , Aktağ, I. G. , Dodig, D. , & Gökmen, V. (2021). Investigations on the formation of Maillard reaction products in sweet cookies made of different cereals. Food Research International, 144, 110352. 10.1016/j.foodres.2021.110352 34053545

